# Empathy training via Kalamazoo Consensus in remote and in-person medical communication: A randomized controlled trial

**DOI:** 10.1016/j.pecinn.2025.100399

**Published:** 2025-05-15

**Authors:** Giovan Battista Previti, Carlo Mazzatenta, Tommaso Bellandi, Francesco Niccolai, Dario Nieri, Valentina Ungaretti, Irene Cavasini, Alessandra Mazzoni, Stefano Maiorano, Luca Di Paolo, Veronica D'Elia, Monica Torre, Licia Matteucci, Guido Miccinesi, Moreno Marcucci, Michela Maielli, Sergio Ardis

**Affiliations:** a North-West Healthcare Public Body, Tuscany, Italy; b Kalamazoo Consensus Statement, Italy; c University Hospital of Pisa, Tuscany, Italy; d Freelancer Dentist, Tuscany, Italy; e Institute for Oncology Study, Prevention and Network, Tuscany, Italy; f Tuscany Regional Government, Tuscany, Italy

**Keywords:** Empathy, Medical education, Communication skill, Kalamazoo consensus statement, Training

## Abstract

**Background:**

Empathy is crucial in healthcare, facilitating effective communication and improving patient outcomes.

**Objective:**

This study aimed to evaluate the impact of tele-conference training based on the Kalamazoo Consensus Statement (KCS) on the empathy scores of newly hired physicians in a tele-visit simulation course.

**Methods:**

From September 2021 to April 2022, we conducted a randomized controlled trial involving 129 medical doctors from 13 hospitals in north-western Tuscany, with an age range of 31 to 42 years. Partecipants were randomly assigned to a trained group (TG) or a control group (CG). Both groups completed the Toronto Empathy Questionnaire (TEQ) and the Balanced Emotional Empathy Scale (BEES) before (T0) and after (T1) the training. The TG underwent a 12-h online communication training course. The CG only completed the questionnaires without further intervention.

**Results:**

Total sample *included 129 partecipants.* Results indicated a significant increase in TEQ scores for the TG (55,8 % of total sample; T0: 65.32; T1: 66.42, *p* = 0.032) and BEES scores (T0: 122.39; T1: 127.50, *p* = 0.000). The CG (44,2 %) experienced a decrease in TEQ scores (T0: 65.58; T1: 63.75, p = 0.000) but stable BEES scores (T0: 122.16; T1: 120.67, *p* = 0.317). Female participants consistently exhibited higher empathy scores than males, with training significantly enhancing scores for both genders.

**Conclusions:**

The tele-conference training effectively improved empathy scores among newly hired physicians. We recommend the implementation of KCS-based training to enhance empathy and communication skills in medical practice.

**Innovation:**

The pandemic has accelerated the use of tele-education and telemedicine, though opinions on their effectiveness remain divided. However, studies show that empathy can be enhanced through interactive online training, which offers significant innovations for both healthcare professionals' learning and patient care.

## 1 Background

Empathy is a multi-dimensional concept [1]: although there is no international consensus, empathy is considered to be the backbone of the patient-physician relationship and clinical care [2].

In Italian Dictionary of Narrative Medicine, empathy is defined as “a behavior generated by a cognitive condition that includes thinking skills, emotional attitudes and the ability to distinguish one's Self from the other in the relationship, capable of producing positive effects on both parties of the relationship” [3].

Empathy is crucial for medical and healthcare practice. It is a fundamental element of patient-centered communication [4], enhancing patient and caregiver satisfaction [[5], [6], [7], [8], [9], [10]], reducing patient anxiety and distress [11,12] and improving physical health outcomes [[13], [14], [15], [16], [17]].

Physician communication is significantly positively correlated with patient adherence [5,[17], [18], [19], [20], [21], [22]]. Empathy and good patient-physician communication skills decrease the risk of patient complaints and litigation [[23], [24], [25], [26]]. Moreover empathy can protect from burnout [27].

Women are more empathic than men in studies on caregivers [[28], [29], [30], [31], [32], [33], [34], [35], [36]]. However, empathy is not genetically inherited [37] and can change throughout our life. Many literature reviews demonstrate the effectiveness of communication training in increasing empathy among healthcare staff [[38], [39], [40], [41]]. Specifically, these studies show that specific training methods were more effective and that personalized feedback improves training more than collective feedback.

In May 1999, 21 leaders and representatives from major medical education and professional organizations in North America attended a conference in Kalamazoo, Michigan: the result was Kalamazoo Consensus Statement (KCS) [42] which identified seven essential elements of physician-patient communication and emphasized the importance of task-focused learning to provides a sense of purpose in acquiring communication skills. These essential elements enhance the effectiveness of physician-patient communication in various practice settings, aiming for patient-centered communication. The Kalamazoo scale has been used to create educational tools and assessment frameworks for learners in different courses such as medical students [[43], [44], [45], [46], [47]], residents [[48], [49], [50], [51]], physicians [49], pediatric medicine [52,53], dentists [54], radiologists [[55], [56], [57]], cardiopulmonary arrest in pediatrics [58], undergraduate nursing students [59], communication in Emergency Department [60], end-of-life communication [61], communication with surrogate decisione-makers [62]. KCS was also used for multi-disciplinary education in a study involving physicians, nurses and a chaplain [49]. In a study on hospitalist-patient communication, KCS was used bedside [63].

Two years of the pandemic may have weakened the empathy of healthcare professionals making it essential to teach or refresh these skills using evidence-based education [64]. Furthermore, it is important to remember that empathic abilities are not static, but can change over time. One of the most studied variations is the loss of empathy that typically occurs in the third year of medical school or other healthcare professions [[29], [30], [31], [32],^34^,^35^,[65], [66], [67]]. Moreover, the rising use of both tele-education and televisits requires new types of teaching and assessment. The aim of our study is to evaluate the impact of tele-conference training on empathy scores of participants in a tele-visit simulation course.

Our study group discussed the term ‘empathy’; in this study, it is used in a broad sense because we concluded that our approach to teaching would not change if we were to offer a training course to non-healthcare professionals. Therefore, the theoretical distinction of empathy has limited educational relevance [[68], [69], [70]].

## 2 Methods

### 2.1 Study design and partecipants

From September 2021 to April 2022, we conducted a randomized controlled trial involving permanent or fixed-term contract medical doctors, recently hired (from 1st June 2019 to 22nd March 2021) in 13 hospitals in the north-west area of Tuscany. We excluded physicians born before 1979 resulting in an age range between 31 and 42 years. This age limit was set to optimize the company's limited resources: the course was reserved for newly hired doctors born after 1979 in order to maximize the data obtained, excluding older doctors who may not have been inclined to modify their communication behaviors.

Due to the narrow age range, stratification of the sample by age was not necessary. The sample was divided by gender and then an online application generated a random list of numbers to assign participants to either a “trained group” (TG) or a “control group” (CG).

### 2.2 Study tool

Both groups were asked to complete two specific empathy questionnaires: the Italian versions of the Toronto Empathy Questionnaire (TEQ) [63,64] and the Balanced Emotional Empathy Scale (BEES) [73,74]. We chose the aforementioned questionnaires because they are the most widely used in Italy and because they are not specific to healthcare professionals.

The BEES, consisting of 30 items, measures the affective or emotional dimension of empathy, which refers to a person's ability to experience emotions similar to those of another individual [86]. The TEQ, consisting of 16 items, explores emotional aspects, primarily focusing on individuals' reactions to the experiences of others, but also the cognitive aspects of empathy [71,72].

The scales were scored online in a form developed on Jotform® which also included fields for biographical (age and gender) and professional information (specialty).

### 2.3 Data collection methods and procedures

Immediately after completing the questionnaires (T0) all participants were asked to conduct a 45-min online visit with a doctor or a nurse specifically trained following the standards for Good Practice of the Association of Standardized Patient Educators [75].

After completing the visit, participants enrolled in the TG were given personalized feedback by one of us and invited to follow the course on doctor-patient communication as specified below.

Participants in the CG were not given any feedback on the simulated tele-visit and were only asked to complete the empathy questionnaires again 4–6 week later (T1). Subsequently these doctors also had the opportunity to follow the doctor-patient communication course on a voluntary basis.

The doctor-patient communication course consists of 12 h of online classroom training delivered in three 4-h sessions over three weeks. The training included lectures, video sessions and debates. The course starts with participants introduction and a brainstorming session to discuss the benefits and challenge of good communication between healthcare professional and patient. A model summarizing the challenges collected in similar brainstorms sessions from previous courses is then discussed. Subsequently the 7-elements of the KCS [42] and other evidence-based communication model involving role-plays and individual feedback [38] are introduced. The Kalamazoo Essential Elements Communication Checklist Adapted (KEECCA) is also explained and used as a teaching tool during the course including a version adapted for tele-visits [76].

Following this we present literature evidence on tasks involved in the interview opening such as self-introduction, the effects of patient interruptions and spontaneous speech time, as well as the benefits of specifically asking the patients about other medical issues they wish to present. The advantages of these practices in terms of rationale time management are also discussed.

A highly interactive one-hour lesson, is dedicated to the use of the para-verbal communications skills during in person visit and tele-visit in relation to the elements of the KCS. Another lesson addresses communication during adverse events. The remainder of the course focuses on empathy and understanding the patient's point of view to point at how to shift from a disease-centered to a patient-centered approach. In the discussion of empathy, the goals are to define what empathy is and explain it in terms of neurobiology, explore how it can be learned and trained, discuss its protective effect against burnout, present the compassion fatigue model [77], and discuss gender differences and the loss of empathy during medical studies.

After completing the course participants in the TG were asked to perform a new simulated tele-visit and asked to fill out again both empathy questionnaires (T1).

All participants were asked to give their consent for the use of their questionnaire results for research purposes.

Expert teachers and simulated patients conducted the training sessions and feedback, as this program is part of a wider initiative to promote effective clinical communication. At the time of the article's submission, 568 healthcare workers had completed the course.

### 2.4 Statistical analysis

Data derived from the two empathy scales were analyzed using Minitab®. The sociodemographic characteristics of the participants (age, gender) were described using descriptive statistics (mean, standard deviation, frequencies and percentages). For data comparison and analysis, paired *t*-tests, independent t-tests and Pearson correlation were used. For data visualization, line plots, interval plots, matrix plots, and power curves were utilized. The α level set at 0.05.

We did not collect any further personal data other than gender and age, in order to facilitate participation in the study. Regarding income, we can state that, although there were some minor differences, the participants were all young public employees, and therefore we could not identify any differences that would have allowed us to stratify the sample.

### 2.5 Institutional review board

The study data were collected as part of the training project to assess course's impact, and we subsequently decided to proceed with publication. Participants freely agreed to data collection and provided consent for the use of data to evaluate the course's effectiveness. Additionally, each participant gave consent for the study at the time of simulation registration (both simulation for the trained group and single one for the control group), and these consents were video-recorded at the start of each video recording. Participants, who were always connected from their home or workplace, were never exposed to any risk resulting from the training activities. The collected data were managed in compliance with current European regulations.

## 3 Results

The study population included 129 physicians (total sample): 72 partecipants (55,8 % of total sample; 51 females and 21 males; mean age 36.4 +/− 2.4 years) were included in the TG, while the remaining 57 (44,2 % of total sample; 38 females and 19 males; mean age 36.5 +/− 2.6 years) constituted the CG.

**Fig. 1:**
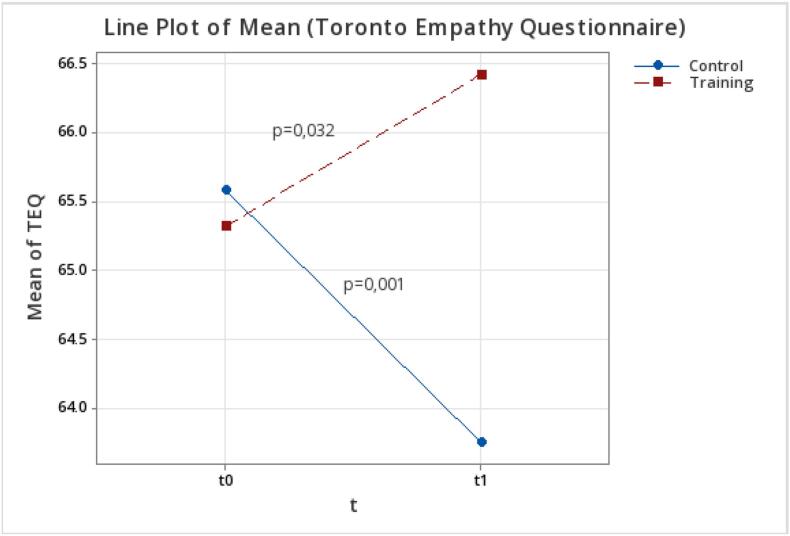
Difference in mean empathy scores for the trained group (TG) and the control group (CG) at T0 and T1 using the TEQ.

Using the TEQ scale (Fig. 1), the mean empathy score for the TG at T0 was 65.32 (SD 5.08) and 66.42 (SD 5.83) at T1 (*p* = 0.032). The CG had a mean empathy score of 65.58 (SD 5.00) at T0 and 63.75 (SD 5.07) at T1 (*p* = 0.000).

**Fig. 2:**
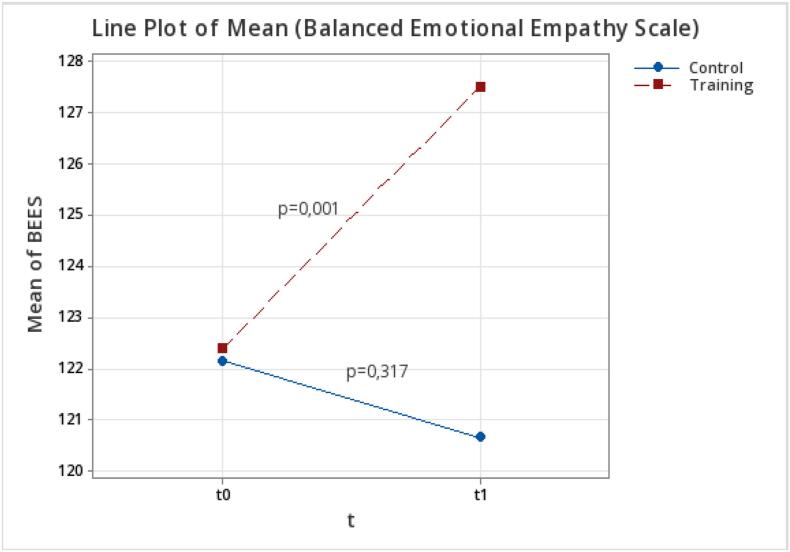
Difference in mean empathy scores for the trained group (TG) and the control group (CG) at T0 and T1 using the BEES.

The mean empathy scores obtained with the BEES (Fig. 2) by the TG were 122.39 (SD 21.17) at T0 and 127.50 (SD 21.53) at T1 (p = 0.000) while the CG scored 122.16 (SD 18.79) at T0 and 120.67 (SD 19.10) at T1, with no statistically significant variation (*p* = 0.317).

In a secondary analysis, we observed that at T0, the mean empathy scores for males were lower than those for females on both the TEQ and BEES questionnaires. After training, males showed a non-significant increase in the TEQ scores but a clear and statistically significant rise in the BEES scores. At T0, females had higher scores compared to males, and following training, they demonstrated a statistically significant increase on both questionnaires. Interestingly, despite the training, the differences between males and females persisted, with trained males reaching only the baseline (T0) empathy scores of both the trained and untrained female groups (Table 1).

**Table 1:** Mean empathy scores in the trained (TG) and control (CG) groups at T0 and T1 divided by gender and *p* value.

		T0	T1	p
TEQ	Male Trained	63,62 SD 5,18	64,29 SD 6,73	0,580
	Male Control	63,16 SD 4,59	61,32 SD 4,91	0,041
	Female Trained	66,02 SD 4,92	67,29 SD 5,24	0,018
	Female Control	66,79 SD 4,81	64,97 SD 4,76	0,004
BEES	Male Trained	106,67 SD 16,73	114,76 SD 17,57	0,012
	Male Control	108,84 SD 17,16	106,74 SD 17,05	0,442
	Female Trained	128,86 SD 19,44	132,75 SD 20,94	0,009
	Female Control	128,82 SD 15,95	127,63 SD 16,16	0,513

As for professional information, almost all medical specialties within our company's population were represented, and it was not possible to perform significant statistical analysis due to the small size of the groups into which the sample could be divided. In any case, we divided the specialties into two groups (technical and non-technical) and found no significant differences. We decided not to report the data also due to the arbitrariness of defining whether a specialty is technical or non-technical.

## 4 Discussion and conclusion

### 4.1 Discussion

Empathy is now regarded as one of the most essential skills in medical practice, as it fosters more meaningful medical interactions, facilitates a shift from a disease-centered to a patient-centered approach, and helps protect healthcare workers from burnout [[79], [80], [81]]. Research indicates that empathy is not genetically determined; it is partially influenced by gender, varies over time, and, most importantly, can be taught and developed. In our study, we demonstrated that a standardized communication course based on the KCS significantly increased empathy scores, as measured by both the TEQ and BEES. In contrast, the untrained group showed stable scores on the BEES and a significant decrease in empathy scores when measured with the TEQ. These findings align with existing literature and confirm that communication courses are effective in enhancing participants' empathy [[38], [39], [40], [41]]. However, to the best of our knowledge, there are no other controlled studies demonstrating the effectiveness of a training course conducted entirely via teleconference, which offers clear advantages in terms of cost reduction, ease of delivery, and the ability to enroll participants from remote locations.

The literature provides numerous evidences demonstrating the effectiveness of empathy and person-centered communication. Empathy improves physical health outcomes [[13], [14], [15], [16]], increases patient satisfaction [9,10], with patients tending to prefer empathetic healthcare providers, recommending them to family and friends [82]. Additionally, empathy enhances patient trust in healthcare providers [83,84], reduces patient anxiety (84, 11), and improves their well-being, in accordance with their individual conditions, when person-centered communication is applied (84). Lastly, it promotes better adherence to treatment [19].

Adopting an empathetic approach also brings practical benefits in clinical practice and in managing professional conflict: it reduces the risk of making errors [25], likely because healthcare providers can better identify patients' symptoms [85], and helps prevent conflicts through empathetic communication [26].

For this reason, it is especially important for young doctors to study and improve their empathetic behavior, an objective that can be easily achieved through teleconference-based training courses.

We also confirmed existing literature showing that women tend to exhibit higher empathy levels, which, in our study, could be further and significantly enhanced by our training programme. Additionally, we demonstrated that while males have lower baseline empathy levels, these can be effectively increased through training, reaching at least the baseline level of our female group.

This data raises the question of whether tailored training should be designed specifically for males, or if we should simply accept that, due to evolutionary and/or cultural factors, females tend to have higher average empathy levels. We are aware of the low power of the test for the male group with both scales used, but we have included the data nonetheless because it aligns with the literature.

Another issue arises from the fact that this study relied on self-reported measures of empathy, which come with the typical limitations of such assessments. Future studies should aim to measure physicians' empathy levels using scales completed by patients during simulated or real clinical encounters, whether in outpatient or tele-visit settings, to confirm that communication courses can have a tangible impact on physician behavior.

### 4.2 Innovation

Although the pandemic has encouraged the use of tele-education and telemedicine, opinions on these two innovative approaches to training healthcare professionals and delivering care remain quite controversial. It is not uncommon to encounter opinions from individuals nostalgic for in-person training, who theoretically question the effectiveness of distance learning. Along the same lines, there are also criticisms regarding the potential to develop empathy during a teleconsultation [78].

Our study, however, has demonstrated that it is possible to enhance empathy through online training courses designed to maximize interaction between learners and with the instructor. Our experience combines tele-education and telemedicine, two frontiers of training and care that today represent a significant innovation with practical and economic implications for both healthcare professionals' learning processes and patients' care processes.

Tele-education and telemedicine represent innovative modalities that require effectiveness measurements to evaluate the various methodologies applicable in both educational and clinical settings.

## 5 Conclusion

The study demonstrated the effectiveness of a teleconference training course based on the Kalamazoo Consensus Statement (KCS) in significantly increasing empathy scores among newly hired physicians, compared to an untrained control group. Empathy declines during the undergraduate degree and specialization, with medical trainees showing lower levels of empathy compared to specialists. Newly hired young doctors need to regain good levels of empathy as early as possible in their professional careers.

However, the development of empathy is often hindered by limiting beliefs, such as the assumption that people already understand empathy and do not need to learn more. Some even believe empathy cannot be learned or view it as potentially harmful, fearing it could diminish a professional's critical judgment. Therefore, we propose that KCS-based training should be implemented for newly hired physicians to enhance empathy and counteract these barriers to its development.

## Limitations of the study

One of the limitations of this study is the small size of the male sample, which restricts the gender analysis. Additionally, while the study measured immediate changes in empathy levels, it does not provide information on the persistence of these changes, which may be temporary. The inclusion of demographic data (such as marital status and offspring) and work-related data (including medical specialty and work setting) also limits the study. Furthermore, changes in empathy levels observed in the study may not necessarily reflect changes in behavior during simulations or in clinical practice.

## CRediT authorship contribution statement

**Giovan Battista Previti:** Writing – review & editing, Supervision, Investigation, Data curation. **Carlo Mazzatenta:** Writing – review & editing, Supervision, Investigation, Data curation. **Tommaso Bellandi:** Writing – review & editing, Supervision, Investigation, Data curation. **Francesco Niccolai:** Writing – review & editing, Supervision, Data curation. **Dario Nieri:** Writing – review & editing, Supervision, Investigation, Data curation. **Valentina Ungaretti:** Writing – review & editing, Supervision, Investigation, Data curation. **Irene Cavasini:** Writing – review & editing, Supervision, Investigation, Data curation. **Alessandra Mazzoni:** Writing – review & editing, Validation, Investigation, Data curation. **Stefano Maiorano:** Writing – review & editing, Supervision, Investigation, Data curation. **Luca Di Paolo:** Writing – review & editing, Supervision, Investigation, Data curation. **Veronica D'Elia:** Writing – review & editing, Supervision, Investigation, Data curation. **Monica Torre:** Writing – review & editing, Supervision, Investigation, Data curation. **Licia Matteucci:** Writing – review & editing, Supervision, Investigation, Data curation. **Guido Miccinesi:** Writing – review & editing, Supervision, Investigation, Data curation. **Moreno Marcucci:** Writing – review & editing, Supervision, Investigation, Data curation. **Michela Maielli:** Supervision, Investigation, Data curation. **Sergio Ardis:** Writing – review & editing, Writing – original draft, Supervision, Investigation, Formal analysis, Data curation.

## Declaration of competing interest

The authors declare that they have no known competing financial interests or personal relationships that could have appeared to influence the work reported in this paper.
